# Cholesterol metabolic rewiring shapes immune remodeling across hepatocarcinogenesis

**DOI:** 10.3389/fimmu.2026.1884948

**Published:** 2026-07-08

**Authors:** Wentao Ma, Fengxu Yan, Yu Cheng, Guoqing Zhang, Yanxi Mu, Zhiqiang Zhang, Xuefeng Liang, Jianxin Gan, Wenwei Yang, Weixiong Zhu, Yusheng Cheng

**Affiliations:** 1 The Second Clinical Medical College, Lanzhou University, Lanzhou, Gansu, China; 2 Nanchang University Queen Mary School, Nanchang, China; 3 Department of General Surgery, The Second Hospital of Lanzhou University, Lanzhou, Gansu, China; 4 Zhangzhou Yuanshan Hospital, Zhangzhou, Fujian, China

**Keywords:** cholesterol metabolism, hepatocellular carcinoma, immunometabolism, MASLD/MASH-HCC, oxysterols, spatial omics, therapeutic resistance, tumor immune microenvironment

## Abstract

Cholesterol metabolism, hepatocellular carcinoma (HCC), and the tumor immune microenvironment are increasingly recognized as interconnected drivers of metabolic dysfunction-associated steatotic liver disease/metabolic dysfunction-associated steatohepatitis-related HCC (MASLD/MASH-HCC). However, cholesterol dysregulation during hepatocarcinogenesis is often discussed as isolated pathways or single-stage events, and evidence strength differs across human HCC tissues, preclinical HCC models, and non-HCC systems. This review integrates mechanistic, spatial multi-omics, and translational evidence to highlight cholesterol dyshomeostasis as a stage- and cell-type-specific rewiring of synthesis, uptake, esterification, efflux, and conversion rather than a uniform metabolic increase. In chronic metabolic liver disease, sterol regulatory element-binding protein 2 (SREBP2)–SREBP cleavage-activating protein (SCAP) activation, impaired bile acid–farnesoid X receptor (FXR) feedback, free-cholesterol loading, and oxysterol accumulation may connect hepatocyte stress with stellate-cell activation, macrophage remodeling, inflammation, and fibrosis. During preneoplastic transition and early HCC, squalene epoxidase (SQLE), sterol O-acyltransferase 1 (SOAT1), farnesyl-diphosphate farnesyltransferase 1 (FDFT1), 24-dehydrocholesterol reductase (DHCR24), and SCAP-regulatory circuits may support membrane remodeling, oncogenic signaling, metabolic autonomy, and impaired immune surveillance, although their evidence levels vary. In advanced and metastatic HCC, spatially resolved studies suggest cholesterol-active tumor regions may be coupled to exhausted T cells, tumor-associated macrophages (TAMs), myeloid-derived suppressor cells (MDSCs), extracellular vesicle signaling, and oxysterol-mediated communication. We further discuss stage-aligned diagnostic and therapeutic opportunities, proposing cholesterol metabolic rewiring as a hypothesis-generating and partially validated framework for HCC initiation, progression, recurrence, and therapeutic resistance.

## 1 Introduction

Hepatocellular carcinoma (HCC) remains a major cause of cancer-related mortality worldwide, but the etiologic landscape of HCC is changing. Hepatitis B virus (HBV) and hepatitis C virus (HCV) continue to be major global drivers, whereas metabolic dysfunction-associated steatotic liver disease (MASLD) and its inflammatory subtype, metabolic dysfunction-associated steatohepatitis (MASH), are emerging as rapidly growing causes of HCC in parallel with obesity, diabetes, and metabolic syndrome (1, 2). This shift makes MASLD/MASH-HCC a particularly important setting in which to understand how metabolic stress, lipid remodeling, inflammation, fibrosis, and immune escape converge during hepatocarcinogenesis. Cholesterol is central to this process because it participates not only in membrane structure and lipid-raft signaling but also in bile acid synthesis, oxysterol generation, organelle stress, stromal activation, and immune-cell function (3–5).

The importance of cholesterol metabolism—and lipid metabolism more broadly—in hepatocellular carcinoma is increasingly recognized. Existing studies have examined the role of SQLE, a key enzyme in cholesterol biosynthesis, in the context of immunotherapy for advanced liver cancer, and others have explored how inhibition of cholesterol-biosynthetic enzymes under high-fatty-acid conditions influences HCC progression (6, 7). However, most of these studies focus on advanced-stage HCC, selected HCC subtypes, or single mechanisms under specific metabolic conditions. Moreover, the spatiotemporal heterogeneity of cholesterol metabolism within HCC tissues remains incompletely defined. The emergence of single-cell and spatial multi-omics technologies now makes it feasible to chart dynamic atlases of disease evolution at cellular resolution (8). These advances create an unprecedented opportunity to systematically define cross-stage trajectories of cholesterol metabolism and spatial microenvironmental crosstalk across the continuum from normal liver to MASH and, ultimately, HCC.

To avoid overinterpretation across heterogeneous experimental systems, this review distinguishes four levels of evidence throughout the text. First, direct human HCC evidence refers to findings derived from human HCC tissues, spatial multi-omics datasets, clinical cohorts, or patient-derived samples. Second, preclinical HCC evidence refers to mechanistic observations from HCC cell lines, organoids, xenografts, genetically engineered mouse models, or diet-induced MASH-HCC models. Third, supporting evidence from non-HCC tumors or general immunology is used only to provide biological plausibility and is not presented as HCC-specific proof. Fourth, mechanistic inferences that remain insufficiently validated in human HCC are explicitly described as hypotheses using terms such as “may suggest,” “is hypothesized to,” “could contribute to,” or “remains to be validated in human HCC.” Guided by the disease-evolution timeline and the spatial architecture of the tumor microenvironment, this review outlines cholesterol homeostasis in the normal liver and then integrates evidence from chronic liver disease, preneoplastic transition, early HCC, and advanced/metastatic HCC. Particular emphasis is placed on distinguishing direct human HCC evidence from preclinical or extrapolative evidence and on linking each metabolic abnormality to a plausible molecular axis and immune or stromal consequence (8–10).

Accordingly, this review has four major objectives. First, we systematically analyze the dynamic features of cholesterol-metabolic reprogramming across the full disease course, from chronic metabolic liver disease and preneoplastic transition to early, advanced, and metastatic HCC. Second, we clarify the molecular mechanisms by which cholesterol-related metabolic disorders reshape immune remodeling, with particular attention to the links among sterol sensing, oxysterol signaling, macrophage polarization, T-cell dysfunction, myeloid suppression, and fibrotic stromal activation. Third, we integrate emerging single-cell and spatial multi-omics evidence to interpret metabolism-immune spatial niches in HCC tissues. Fourth, we propose a stage-aligned framework for biomarkers and therapeutic strategies, aiming to connect cholesterol-related molecular alterations with risk stratification, early detection, recurrence prediction, and rational metabolic-immune combination therapy.

## 2 Cholesterol homeostasis in the normal liver

The liver is the central organ responsible for whole-body cholesterol homeostasis. It not only carries out local processes such as cholesterol synthesis, uptake, esterification, storage, conversion, and transport, but also coordinates systemic cholesterol balance through integrated regulation among hepatocytes and non-parenchymal cells. Through multilayered feedback networks, the liver maintains a dynamic balance between cholesterol supply and demand, thereby preserving metabolic homeostasis.

Within the liver, hepatocytes are the principal effector cells of cholesterol metabolism. Hepatic cholesterol handling can be broadly organized into five interconnected processes: synthesis, uptake, esterification and storage, conversion and excretion, and efflux. *De novo* cholesterol synthesis in hepatocytes is primarily regulated by the SREBP2-dependent mevalonate pathway in the endoplasmic reticulum. When intracellular sterol levels fall, SREBP2 activates the rate-limiting enzyme 3-hydroxy-3-methylglutaryl-coenzyme A reductase (HMGCR) and simultaneously upregulates low-density lipoprotein receptor (LDLR) expression, thereby enhancing LDL-derived cholesterol uptake (11, 12). Under conditions of cholesterol excess, free cholesterol can be esterified by sterol O-acyltransferases (SOAT/ACAT enzymes, including SOAT1/ACAT1 and SOAT2/ACAT2) into cholesteryl esters for storage, helping buffer excess free cholesterol and limit lipotoxicity (13).

Cholesterol disposal in hepatocytes occurs predominantly through bile acid synthesis and biliary secretion. In the classical bile acid synthesis pathway, cholesterol 7-hydroxylase (CYP7A1) is the rate-limiting enzyme. Its activity is tightly restrained by bile acid-mediated negative feedback, mainly through the hepatic FXR-SHP pathway and the enterohepatic FXR-FGF19 axis, thereby preventing excessive bile acid accumulation (14). Hepatocytes also participate in reverse cholesterol transport through cholesterol-efflux pathways. In this context, ABCA1 mainly mediates cholesterol efflux to lipid-poor apolipoprotein A-I (apoA-I) and is essential for nascent HDL formation, whereas ABCG1 mainly promotes cholesterol efflux to mature HDL particles rather than to apoA-I (15). Coordinated regulation across these processes collectively maintains hepatic cholesterol homeostasis (11, 12, 14, 15).

Beyond hepatocytes, non-parenchymal liver cells also contribute substantially to hepatic cholesterol homeostasis. As liver-resident macrophages, Kupffer cells internalize modified lipoproteins such as oxidized LDL (oxLDL) predominantly through scavenger receptors, including SR-A/MSR1 and CD36, whereas cholesterol derived from apoptotic cells is acquired mainly through efferocytosis rather than through LDLR-dependent uptake (16, 17). Excess intracellular cholesterol can subsequently be esterified as a buffering mechanism and can also be removed through liver X receptor (LXR)-dependent ABCA1/ABCG1-mediated efflux pathways, thereby supporting local cholesterol balance and reverse cholesterol transport (17).

Hepatic stellate cells (HSCs) should not be described simply as cells that primarily store cholesterol. In the quiescent state, HSCs are specialized lipid-droplet-rich cells that primarily store retinoids while also containing other neutral lipids, including triglycerides and cholesteryl esters (18–20). During HSC activation, free cholesterol accumulation promotes fibrogenic responses by enhancing TLR4 signaling, downregulating BAMBI, and sensitizing HSCs to TGF--mediated activation (21). In this setting, ACAT1/SOAT1 serves a protective buffering role against free-cholesterol accumulation, and disruption of this mechanism aggravates liver fibrosis (22). Therefore, HSCs play an important and context-dependent role in cholesterol-related fibrogenesis (18, 21, 22).

Liver sinusoidal endothelial cells (LSECs) regulate hepatic cholesterol metabolism mainly by maintaining sinusoidal exchange and by providing angiocrine signals that sustain hepatocyte metabolic zonation. LSEC-derived Wnt ligands, particularly Wnt2 and Wnt9b, are important for maintaining -catenin-dependent pericentral metabolic programs in hepatocytes (23, 24). In humanized liver models, endothelial WNT2 has been shown to regulate hepatocyte cholesterol uptake and bile acid conjugation through FZD5, indicating an important role for endothelial–hepatocyte crosstalk in hepatic cholesterol handling (25). In addition, the fenestrated structure of LSECs facilitates the transfer of lipoproteins and other solutes between sinusoidal blood and hepatocytes, and LSECs also participate in the clearance of circulating oxLDL through scavenger functions involving stabilin-1 and stabilin-2 (26). Accordingly, LSECs are best understood as supporting hepatic cholesterol trafficking, zonated metabolic regulation, and lipoprotein clearance, whereas LSEC dysfunction or capillarization may impair these processes and contribute to MASLD/NASH progression (27, 28).

## 3 Cholesterol metabolic dysregulation during chronic liver disease (MASLD/MASH and fibrosis)

**Figure 1:**
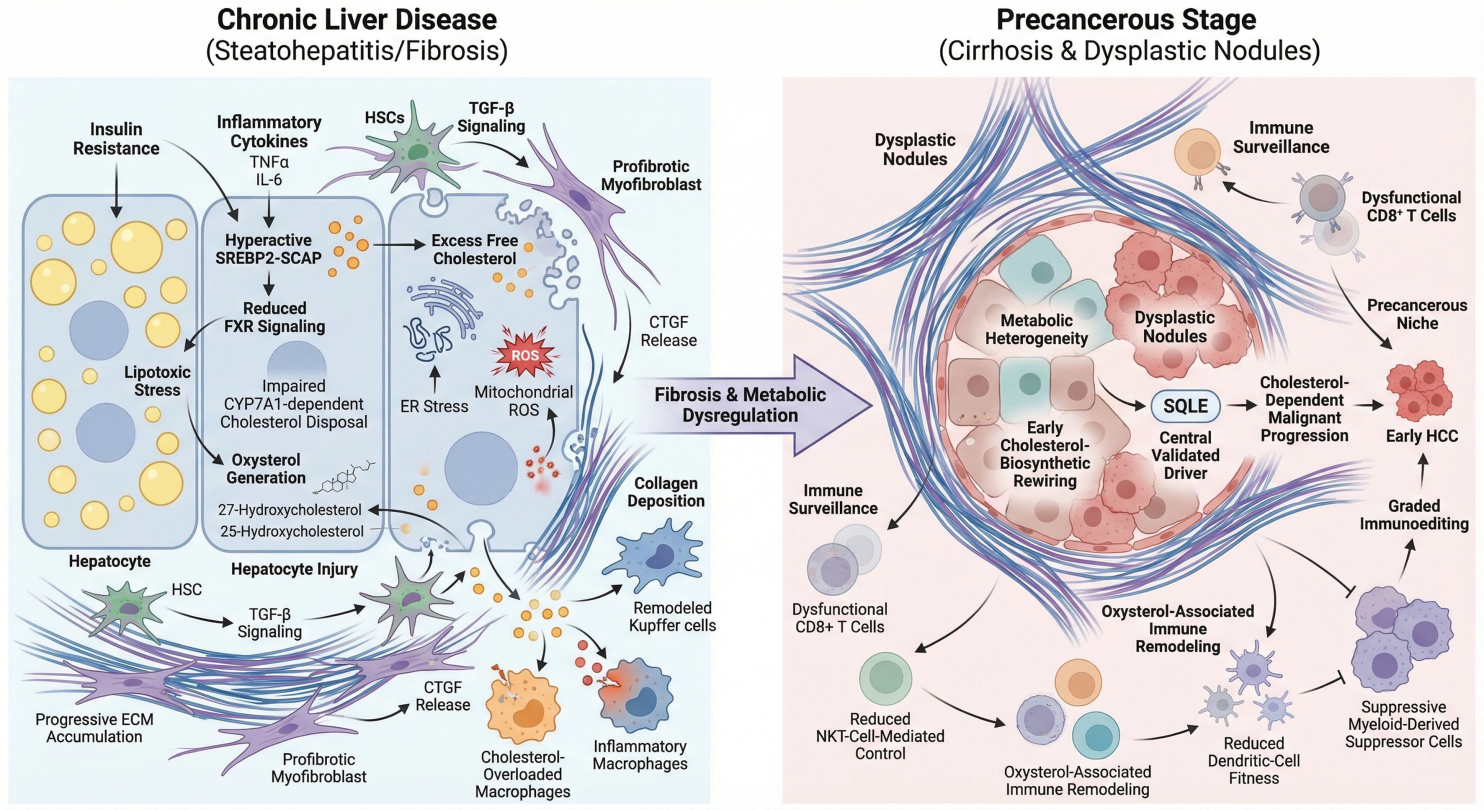
Cholesterol metabolic dysregulation shapes a fibrotic and immune-permissive precancerous niche. This image illustrates how chronic liver injury progressively evolves into a precancerous microenvironment through coordinated metabolic, stromal, and immune alterations. In metabolically stressed hepatocytes, insulin resistance, inflammatory signaling, impaired FXR-mediated bile acid feedback, and excessive SREBP2 activation drive cholesterol accumulation, oxysterol generation, ER stress, and lipotoxic injury. These events activate hepatic stellate cells and remodel hepatic macrophages, promoting extracellular matrix deposition and fibrosis. Within this chronically injured background, dysplastic nodules emerge in association with increasing metabolic heterogeneity and cholesterol-biosynthetic rewiring, including progressive reinforcement of SQLE-centered tumor-promoting programs. In parallel, cholesterol and oxysterol imbalance contributes to impaired CD8+ T-cell and NKT-cell surveillance, suppressive myeloid-cell accumulation, and broader immunoediting. FXR, farnesoid X receptor; SREBP2, sterol regulatory element-binding protein 2; ER, endoplasmic reticulum; HSCs, hepatic stellate cells; SQLE, squalene epoxidase; MDSCs, myeloid-derived suppressor cells.

Persistent chronic metabolic stress disrupts hepatic cholesterol homeostasis and drives progression from steatosis to steatohepatitis (traditional terminology, NAFLD → NASH; updated nomenclature, MASLD → MASH). This stage is characterized by increased intrahepatic cholesterol synthesis and uptake, constrained efflux and biotransformation, and aberrant activation of non-parenchymal cells within the hepatic microenvironment. These alterations reinforce one another and establish a pro-inflammatory, profibrotic milieu that provides the pathological basis for subsequent HCC development (**Figure 1**).

### 3.1 Metabolic turning points in hepatocytes

Under metabolic stress, hepatocytes shift from regulators of cholesterol homeostasis toward drivers of metabolic dysfunction. This reprogramming is manifested through a series of interconnected inflection points, including insulin resistance with dysregulated SREBP2-dependent cholesterol metabolism, bile acid imbalance with impaired FXR signaling, and lipotoxic stress accompanied by oxysterol accumulation.

#### 3.1.1 Insulin resistance and hyperactivation of SREBP2

Insulin resistance is a core pathological hallmark of MASLD/MASH and a major driver of hepatocellular lipid-metabolic dysfunction. Mechanistically, nutrient excess, altered sterol sensing, and inflammatory cytokines can converge on the TNF-/IL-6-NF-B-SREBP2 axis, thereby increasing SREBP2 transcriptional activity and promoting HMGCR- and LDLR-dependent cholesterol synthesis and uptake (29–31). However, this pathway is not uniformly injurious in all experimental settings: an Insig1-knockout mouse model showed that suppression of INSIG1 promoted hepatic lipid remodeling and restrained NASH progression rather than aggravating hepatocyte injury (32). These data indicate that SREBP2-SCAP dysregulation may contribute to cholesterol dyshomeostasis in MASLD/MASH, but the direction and magnitude of its effect remain context-dependent and require careful validation in human MASLD/MASH-HCC progression.

#### 3.1.2 Bile acid metabolism dysregulation and impaired FXR signaling

Bile acids constitute a major route of cholesterol disposal, and farnesoid X receptor (FXR), a principal nuclear receptor for bile acids, regulates both cholesterol and bile acid homeostasis. Under physiological conditions, the hepatic FXR-SHP pathway and the intestinal FXR-FGF15/19 axis restrain cholesterol 7-hydroxylase (CYP7A1), thereby maintaining bile acid balance (33–35). In MASLD/MASH, insulin resistance, inflammatory signaling, and altered bile acid pools may weaken this feedback loop, impair cholesterol catabolism, and promote hepatic cholesterol accumulation (36, 37). Loss-of-function studies in FXR-deficient mice support a causal link between impaired FXR signaling, lipid/glucose metabolic disorder, and hepatic inflammation (38). Therefore, impaired FXR signaling may connect cholesterol disposal failure with inflammatory and fibrotic remodeling, although the strength of evidence differs between mouse models and human MASLD/MASH-HCC.

#### 3.1.3 Lipotoxic stress and oxysterol accumulation

Lipotoxic stress and oxysterol accumulation are important but context-dependent contributors to progression from MASLD to MASH (39). Mechanistically, lipid overload induces endoplasmic reticulum (ER) stress, mitochondrial dysfunction, ROS production, and unfolded protein response (UPR) activation; these processes can enhance SREBP2-dependent cholesterol biosynthesis and generate oxysterols such as 25-hydroxycholesterol (25-HC) and 27-hydroxycholesterol (27-HC) (40–42). Oxysterols may subsequently engage LXR-dependent efflux programs as a compensatory response, while also influencing inflammatory and stress signaling in a cell-type-specific manner (42, 43). Thus, lipotoxicity should be interpreted as a metabolic-stress network linking ER stress, oxidative stress, SREBP2 activation, oxysterol generation, and immune/stromal remodeling rather than as a single linear pathway.

### 3.2 The microenvironment of chronic liver disease

In chronic liver disease, dysregulated cholesterol metabolism in lipotoxic hepatocytes may initiate immune and stromal remodeling through defined molecular bridges. Free cholesterol and oxysterols can induce ER stress, ROS production, and inflammatory cytokine release; these hepatocyte-derived stress signals are hypothesized to activate hepatic stellate cells (HSCs), remodel Kupffer-cell/macrophage states, and reinforce fibrosis-associated inflammation. Direct liver-disease evidence supports the role of 27-HC in HSC activation, whereas several macrophage-polarization mechanisms remain supported mainly by preclinical or non-HCC immune models and therefore require validation in human MASLD/MASH-HCC.

#### 3.2.1 Activation of hepatic stellate cells

Hepatic stellate cells (HSCs) play a key role in cholesterol-metabolism dysregulation during chronic liver disease. Direct experimental liver-disease evidence indicates that free-cholesterol accumulation can enhance TLR4 signaling, downregulate BAMBI, and sensitize HSCs to TGF--mediated activation, thereby promoting myofibroblastic transition and extracellular matrix deposition (21, 22). In addition, 27-HC can activate oxidative-stress- and TGF--associated profibrotic signaling in HSCs, supporting a cholesterol/oxysterol-TLR4/TGF--fibrosis axis (44). Activated HSCs may further amplify immune remodeling by secreting TGF-, CTGF, TNF-, IL-1, and IL-6, but the relative contribution of each mediator to human MASLD/MASH-HCC remains to be determined (45, 46).

#### 3.2.2 Remodeling of hepatic macrophages/Kupffer cells

In MASLD/MASH, changes in hepatic macrophage/Kupffer-cell activation states are closely associated with altered cholesterol metabolism (47). Mechanistically, cholesterol overload can activate inflammatory programs such as the C/EBP-VCAM1 axis in Kupffer cells, thereby promoting hepatic inflammation (48). Conversely, 25-HC has been reported in some macrophage models to engage the SCAP-SREBP-1a-LXR axis, promote cholesterol efflux, and restrain M1-like polarization; this represents supporting immune-cell evidence rather than an established MASLD/MASH-HCC-specific mechanism. Other studies suggest that cholesterol metabolites may promote M2-like polarization under selected conditions (49, 50). Together, these divergent observations indicate that macrophage remodeling is driven by cholesterol dyshomeostasis and local inflammatory context, not by a uniform shift toward a single polarization state.

## 4 Coupling of cholesterol metabolic reprogramming and immunoediting during the precancerous stage

During hepatocarcinogenesis, precursor lesions such as dysplastic nodules—particularly high-grade dysplastic nodules arising in cirrhotic livers—develop within a chronically injured and fibrotic microenvironment. Cholesterol-metabolic dysregulation evolves along a continuum from chronic liver disease to early HCC, while immunoediting progressively shapes a permissive microenvironment for malignant transition (**Figure 1**).

### 4.1 Metabolic heterogeneity in dysplastic nodules

Dysplastic nodules (DNs) are recognized preneoplastic lesions in hepatocarcinogenesis and typically develop in the setting of cirrhosis or advanced fibrosis. Although DNs display cytological and architectural atypia, they do not yet meet the diagnostic criteria for HCC. Rather, they represent an intermediate stage between regenerative nodules and overt malignancy (51, 52). Emerging evidence suggests that metabolic heterogeneity begins to emerge during this transitional phase; cholesterol-biosynthetic rewiring may arise in precursor lesions and become progressively reinforced during progression toward early HCC (53).

Squalene epoxidase (SQLE), a rate-limiting enzyme in cholesterol biosynthesis, is now well established as an oncogenic metabolic node in liver cancer. p53 transcriptionally represses SQLE, and p53 loss leads to increased cholesterol biosynthesis and tumor growth. In HCC, SQLE upregulation promotes proliferation, migration, and metastasis and activates TGF-β/SMAD signaling. In MASH-HCC, SQLE also impairs antitumor immunity by attenuating CD8+ T-cell function and increasing immunosuppressive MDSCs, thereby contributing to resistance to anti-PD-1 therapy. Therefore, SQLE should be described as a validated driver of cholesterol-dependent malignant progression in HCC and MASH-HCC, whereas its direct role in dysplastic nodules remains to be more clearly defined (54–56).

### 4.2 Metabolism-driven immunoediting

As liver disease progresses from chronic inflammation and fibrosis toward malignancy, dysregulated cholesterol metabolism increasingly reshapes the immune microenvironment. Current evidence supports a graded model in which cholesterol and oxysterol imbalance, altered sterol sensing, and fibrosis-associated stromal signals may weaken immune surveillance and favor immunosuppressive reprogramming. However, this conclusion draws on different evidence levels: direct HCC evidence is strongest for selected tumor-stage mechanisms, preclinical liver models support several carcinogenesis-related pathways, and some immune-cell mechanisms are extrapolated from broader tumor or immunology studies (56, 57). Therefore, mechanisms involving cytotoxic T cells, natural killer T (NKT) cells, dendritic cells, and myeloid-derived suppressor cells (MDSCs) should be interpreted as stage- and model-dependent rather than uniformly established in all dysplastic nodules.

#### 4.2.1 Dysregulated cholesterol homeostasis and T-cell dysfunction

Cholesterol homeostasis is essential for T-cell activation, membrane organization, and signal transduction. The effect of cholesterol on CD8+ T-cell function is not unidirectional. Supporting evidence from tumor models indicates that cholesterol accumulation can induce ER-stress-associated checkpoint upregulation and exhaustion, whereas recent evidence suggests that oxysterol-driven LXR activation may suppress SREBP2, cause cholesterol deficiency in intratumoral CD8+ T cells, impair mTORC1 signaling, and promote exhaustion-like dysfunction (6, 43, 57–60). These apparently divergent findings suggest that T-cell dysfunction is better conceptualized as cholesterol dyshomeostasis rather than simple cholesterol excess. Whether the same mechanisms operate in human dysplastic nodules before overt HCC remains to be validated.

In parallel, NKT cells, which contribute to hepatic antitumor immunosurveillance, are also vulnerable to cholesterol metabolic disturbance. In obesity-associated NAFLD-HCC models, hepatic cholesterol accumulation selectively suppresses NKT-cell expansion and cytotoxicity, thereby weakening immune surveillance and promoting hepatocarcinogenesis. This supports the broader concept that aberrant cholesterol signaling in the diseased liver can disable lymphocyte-mediated tumor control before or during early malignant transformation (61).

#### 4.2.2 Oxysterol-associated immunosuppressive remodeling

Oxysterols are bioactive cholesterol derivatives that influence immune-cell differentiation, trafficking, and inflammatory responses. In chronically injured livers, disturbed lipid metabolism and oxidative stress may promote oxysterol accumulation. Preclinical liver-tumor evidence shows that chronic LXR activation can sensitize mice to HCC development, at least in part through oxysterol accumulation, expansion of innate immune-suppressor populations, reduced dendritic cells and cytotoxic T cells, and activation of IL-6/JAK/STAT3 and complement-related programs (62, 63). Nevertheless, LXR activation can also promote cholesterol efflux as a homeostatic response, indicating that oxysterol-LXR signaling may have divergent consequences depending on cell type, disease stage, and ligand context (43). Thus, oxysterol-LXR signaling may contribute to immune-permissive remodeling during hepatocarcinogenesis, but its precise role in human precancerous lesions remains to be validated.

#### 4.2.3 Expansion and maintenance of myeloid-derived suppressor cells

Myeloid-derived suppressor cells (MDSCs) are important mediators of immune evasion in HCC. Direct and cross-species HCC evidence supports the enrichment of suppressive myeloid populations in fibrosis-associated HCC, including PPP1R15A-expressing monocytic MDSCs that reflect stromal-myeloid crosstalk (64). Mechanistically, fibrosis-associated cytokines, oxysterol-related metabolic remodeling, and altered lipid handling may enhance MDSC survival, suppressive fitness, and persistence (65, 66). However, cholesterol- and oxysterol-specific mechanisms of MDSC expansion are still incompletely defined in human HCC, and conclusions derived from general tumor immunometabolism should be presented as supporting evidence rather than HCC-specific proof.

## 5 Metabolic reprogramming and immune remodeling in early hepatocellular carcinoma

**Figure 2:**
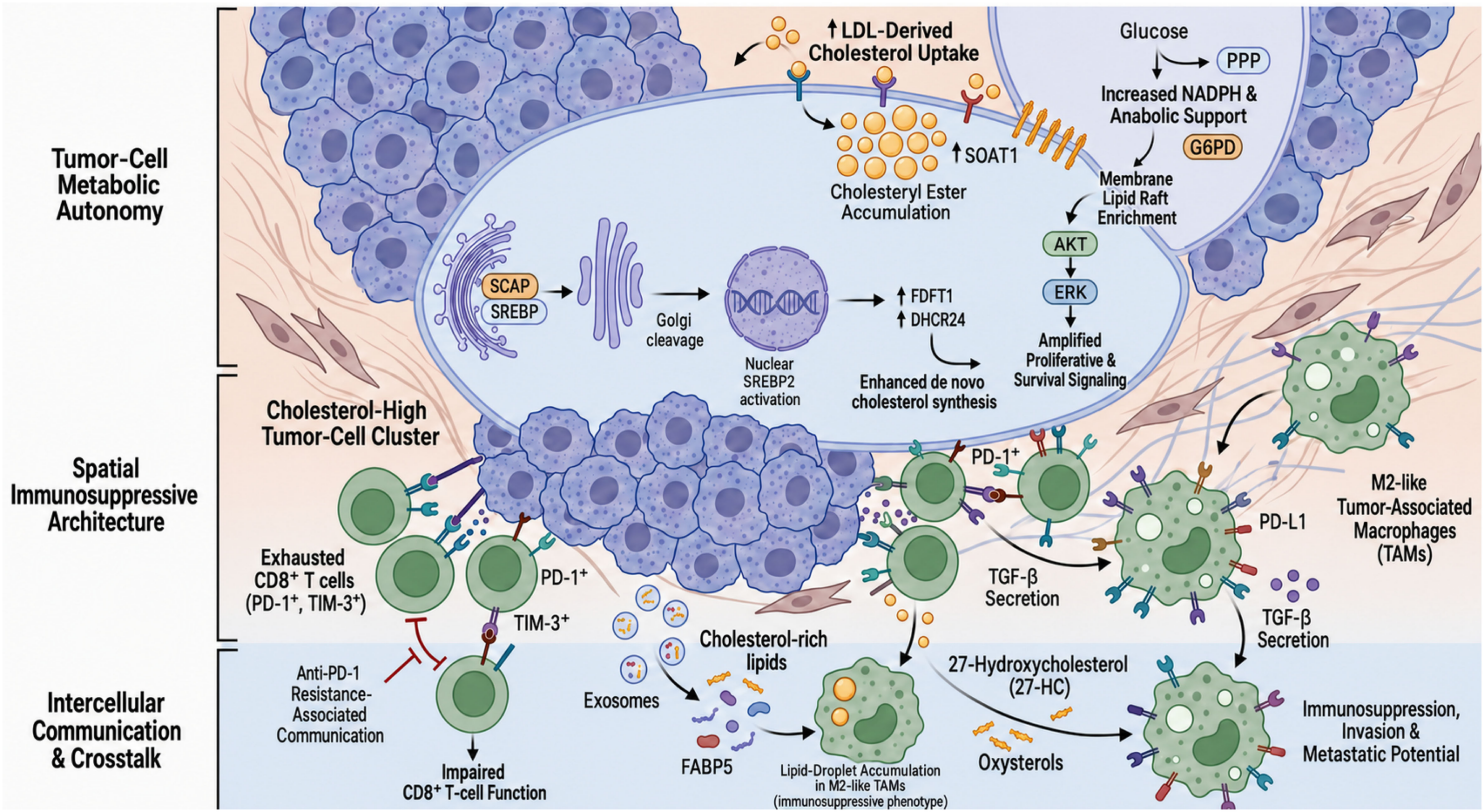
Early HCC is characterized by cholesterol-driven metabolic autonomy, spatial immunosuppression, and lipid-mediated intercellular crosstalk. This schematic illustrates cholesterol-related metabolic and immune remodeling in early hepatocellular carcinoma (HCC). Tumor cells may enhance LDL-derived cholesterol uptake, SOAT1-mediated cholesteryl ester storage, and SCAP/SREBP2-dependent *de novo* cholesterol synthesis through ER-to-Golgi trafficking, Golgi cleavage, and nuclear SREBP2 activation. The PPP/G6PD-NADPH axis further supports lipid biosynthesis and membrane lipid-raft enrichment, thereby potentially amplifying AKT/ERK-mediated proliferative and survival signaling. Spatially, cholesterol-high tumor-cell clusters have been associated in selected HCC contexts with exhausted CD8+ T cells, PD-L1+ M2-like TAMs, and TGF- signaling. Through cholesterol-rich exosomes, FABP5-associated lipid transfer, and oxysterols such as 27-HC, tumor cells may promote macrophage lipid-droplet accumulation, CD8+ T-cell dysfunction, and immunosuppression, although several axes remain to be validated in human early HCC. SOAT1, sterol O-acyltransferase 1; SCAP, SREBP cleavage-activating protein; SREBP2, sterol regulatory element-binding protein 2; PPP, pentose phosphate pathway; G6PD, glucose-6-phosphate dehydrogenase; TAMs, tumor-associated macrophages; FABP5, fatty acid binding protein 5; 27-HC, 27-hydroxycholesterol.

Primary hepatocellular carcinoma (HCC) undergoes coupled metabolic and immune shifts from the earliest stages. By increasing heterogeneity in cholesterol biosynthesis and uptake pathways, tumor cells progressively establish metabolic autonomy; during this process, dynamic shifts in cholesterol distribution and redistribution of membrane cholesterol potentiate proliferative signaling (e.g., the EGFR/PI3K pathway). Concurrently, regions with high cholesterol metabolic activity are often associated with an immunosuppressive microenvironment characterized by T-cell exhaustion and enrichment of M2-like tumor-associated macrophages. The interplay between metabolic reprogramming and immune evasion may contribute to tumor progression, laying a foundation for early HCC development (**Figure 2**).

### 5.1 Establishment of metabolic autonomy in tumor cells

Tumor cells gradually acquire metabolic autonomy during the early stages of HCC, but this process should be defined as a coordinated network rather than a simple increase in cholesterol abundance. Direct and preclinical HCC evidence indicates that cholesterol biosynthesis, LDL-derived uptake, SOAT1-mediated esterification, and SCAP-SREBP2-dependent sterol sensing can cooperate to maintain membrane biogenesis, lipid-raft signaling, and stress buffering. These metabolic changes may then promote EGFR/PI3K/AKT and ERK signaling, epithelial-mesenchymal transition (EMT), and immune escape, although the dominant pathway varies by tumor subtype, etiology, and microenvironmental context.

#### 5.1.1 High expression of key enzymes and regulation of cholesterol metabolism

In early HCC, SOAT1 upregulation may mitigate cholesterol-induced cytotoxicity by converting excess free cholesterol into cholesteryl esters. By reducing the endoplasmic-reticulum-accessible free-cholesterol pool, SOAT1 is hypothesized to facilitate SCAP-SREBP trafficking from the endoplasmic reticulum to the Golgi apparatus, where SREBP2 is proteolytically activated and subsequently translocated into the nucleus. This axis may increase cholesterol biosynthetic and uptake-related genes, including FDFT1, DHCR24, and LDLR, thereby supporting *de novo* cholesterol synthesis, cholesterol uptake, metabolic autonomy, tumor-cell proliferation, invasiveness, and pro-tumor signaling. Because direct evidence that SOAT1 universally activates SCAP-SREBP2 in human early HCC remains limited, this mechanism should be described as a plausible model that requires further validation.

Sterol O-acyltransferase 1 (SOAT1), also known as acyl-CoA: cholesterol acyltransferase 1 (ACAT1), is a key enzyme in cholesterol esterification. Direct HCC evidence indicates that SOAT1 is upregulated in HCC relative to normal liver and that high SOAT1 expression correlates with tumorigenesis and disease progression (67). Preclinical HCC data further show that SOAT1 overexpression promotes lipid-droplet and cholesteryl-ester accumulation, increases ITGAV and ITGB4 expression, promotes EMT, and contributes to HCC invasiveness and metastasis (67). Thus, the better-supported axis is SOAT1-cholesteryl ester storage-integrin/EMT-invasive behavior, whereas the SOAT1-SCAP-SREBP2 feedback model should be retained as a mechanistic hypothesis rather than stated as fully proven in human HCC.

Farnesyl-diphosphate farnesyltransferase 1 (FDFT1) is a pivotal enzyme in cholesterol biosynthesis that catalyzes squalene production. FDFT1 upregulation facilitates HCC progression, whereas FDFT1 suppression lowers intracellular cholesterol and bile acids, activates hepatocyte nuclear factor 4 (HNF4), increases ALDOB expression, inhibits AKT1 phosphorylation, and delays HCC progression through the HNF4A–ALDOB–AKT1 axis (68).

24-dehydrocholesterol reductase (DHCR24; also known as Seladin-1) is essential for cholesterol synthesis, catalyzing the reduction of desmosterol to cholesterol as the terminal step in *de novo* cholesterol biosynthesis. DHCR24 is pro-tumorigenic in HCC, and acetylation at Lys254 (K254) serves as a key metabolic switch that sustains persistent cholesterol biosynthesis while concomitantly driving 7-KC accumulation. 7-KC can stabilize p62 and suppress autophagic flux, thereby facilitating tumor development (69).

#### 5.1.2 Dynamic changes in intracellular cholesterol distribution

By strengthening cholesterol biosynthesis, HCC cells can increase endogenous cholesterol availability for membrane biogenesis and proliferative signaling, but this advantage is context-dependent. Preclinical HCC evidence indicates that zinc finger DHHC-type palmitoyltransferase 3 (ZDHHC3) and abhydrolase domain containing 17A (ABHD17A) dynamically regulate SCAP S-acylation, enhancing SCAP activation and SREBP nuclear entry to increase cholesterol-biosynthetic genes (70). Cholesterol biosynthesis is also metabolically coupled to the pentose phosphate pathway (PPP): PPP-derived NADPH provides reducing power for lipid synthesis, whereas ribose-5-phosphate supports nucleotide biosynthesis; in turn, active sterol synthesis can reinforce anabolic flux (71). This ZDHHC3/ABHD17A-SCAP-SREBP2-PPP/NADPH axis provides a more explicit mechanistic bridge between cholesterol metabolism, proliferative signaling, and immune escape, although its quantitative contribution in human early HCC remains to be further defined.

### 5.2 Cholesterol metabolism-driven spatial immunosuppression and intercellular communication

In HCC, cholesterol-metabolic reprogramming reshapes the immune microenvironment through the dual mechanisms of spatial compartmentalization and intercellular crosstalk. In some HCC contexts, highly metabolic tumor cells form spatial niches enriched for exhausted T cells and pro-tumor macrophages and may disseminate immunosuppressive signals through exosomes and oxysterols.

#### 5.2.1 Spatial distribution effects within the immune microenvironment

In HCC, cholesterol-metabolic reprogramming occurs not only within tumor cells but also reshapes nutrient competition, membrane architecture, and signal transduction between tumor and immune cells. Spatial transcriptomics and high-dimensional imaging provide direct human HCC evidence that lipid/cholesterol-active tumor regions can be located near exhausted CD8+ T cells and pro-tumor macrophages in selected contexts, such as intratumoral steatosis or minimal residual disease (70, 72–76). Mechanistically, membrane-cholesterol enrichment may promote lipid-raft assembly and amplify EGFR/PI3K/AKT and ERK signaling, while PD-L1+ TAM-CD8+ T-cell proximity and TGF--associated macrophage-tumor crosstalk may support local immune suppression and recurrence. These spatial associations are biologically informative but should not be interpreted as proof of direct metabolite transfer without spatial metabolomic or functional validation.

Tumor cells within high-cholesterol-metabolism regions commonly exhibit coordinated upregulation of biosynthetic and uptake programs, featuring an active SREBP-driven mevalonate-cholesterol pathway accompanied by membrane-cholesterol enrichment and enhanced lipid-raft formation. Increased membrane cholesterol and lipid rafts may amplify receptor-associated AKT and ERK cascades, thereby enhancing proliferative and survival signaling (70, 73, 77, 78). However, because the spatial location of these tumor states can vary between the tumor core, invasive front, residual disease, and steatotic regions, the cholesterol-high phenotype should be interpreted as a context-specific spatial niche rather than a universal architectural feature of all HCCs.

cell exhaustion zones adjacent to highly metabolic tumor regions are characterized by suppressed CD8+ T-cell effector functions, persistent high expression of immune checkpoint molecules (e.g., PD-1 and TIM-3), and a discrete clustered distribution (57, 73, 79). Metabolically, T-cell dysfunction is more accurately linked to cholesterol dyshomeostasis than to uniformly increased intracellular cholesterol; recent evidence shows that oxysterol-driven LXR activation with reciprocal SREBP2 suppression can induce cholesterol deficiency in intratumoral CD8+ T cells, impairing mTORC1 signaling and promoting exhaustion-like dysfunction (57, 59, 60).

Regions enriched for M2-like tumor-associated macrophages (TAMs) constitute an important component of this spatial neighborhood. Cholesterol-metabolic reprogramming may shift macrophages toward immunosuppressive states by altering lipid uptake, processing, efflux, and nuclear-receptor signaling; these changes can be accompanied by PD-L1 expression and secretion of TGF-, thereby suppressing CD8+ T-cell cytotoxicity (74, 80–82). Spatial evidence in residual or recurrence-associated HCC suggests that macrophage-tumor crosstalk, particularly through TGF--associated signaling, helps maintain adaptive tumor-cell states and is associated with T-cell exhaustion and recurrence (74). Nevertheless, the direction of macrophage polarization remains context-dependent, and both pro-inflammatory and pro-tumor macrophage programs should be considered.

#### 5.2.2 Intercellular communication

In early-stage HCC, tumor cells often exhibit increased cholesterol synthesis and uptake, but how this metabolic burden is transmitted to neighboring cells remains incompletely defined. Two plausible routes are supported by different evidence levels: cholesterol-rich extracellular vesicles/exosomes can deliver lipid cargo and lipid-handling proteins to recipient cells, whereas oxysterols such as 27-HC can function as soluble immunoregulatory mediators. These routes may convert tumor-cell cholesterol dyshomeostasis into macrophage lipid remodeling, CD8+ T-cell dysfunction, and local immune suppression, but direct causal validation in human early HCC remains limited.

Exosomal membranes are enriched in cholesterol and sphingomyelin, allowing extracellular vesicles to serve as lipid-delivery platforms and possible buffers for excess cholesterol. Direct preclinical HCC evidence shows that HCC-derived exosomes can transfer fatty acid binding protein 5 (FABP5) to macrophages, enhance lipid-droplet accumulation, and induce M2-like changes; these effects are attenuated when exosomal FABP5 is absent (83). This supports a tumor-exosome-FABP5-macrophage lipid remodeling axis. Beyond myeloid cells, HCC-derived exosomes can also induce CD8+ T-cell dysfunction and have been associated with anti-PD-1 resistance (84, 85). However, whether exosomal cholesterol itself is the dominant causal cargo, rather than associated proteins or other lipids, remains to be validated in human HCC.

A parallel route to exosomes is oxysterol efflux. Oxysterols are both metabolic products and immunoregulatory molecules that act on nuclear receptors and chemotactic axes to alter immune-cell migration, differentiation, and effector function. Direct HCC evidence supports a TMEM147-STAT2-DHCR7-27-HC-TAM axis: TMEM147 upregulation activates STAT2 to increase DHCR7 transcription, elevating intracellular and extracellular 27-HC; secreted 27-HC then promotes M2-like TAM polarization and enhances invasion and metastasis (86). By contrast, oxysterol-LXR-mediated impairment of dendritic-cell CCR7 expression and oxysterol-CXCR2-mediated recruitment of pro-tumor neutrophils are supported mainly by broader tumor models and should be presented as biologically plausible but not yet HCC-specific mechanisms (87–91).

## 6 Metabolic adaptation and systemic effects of metastatic hepatocellular carcinoma

**Figure 3:**
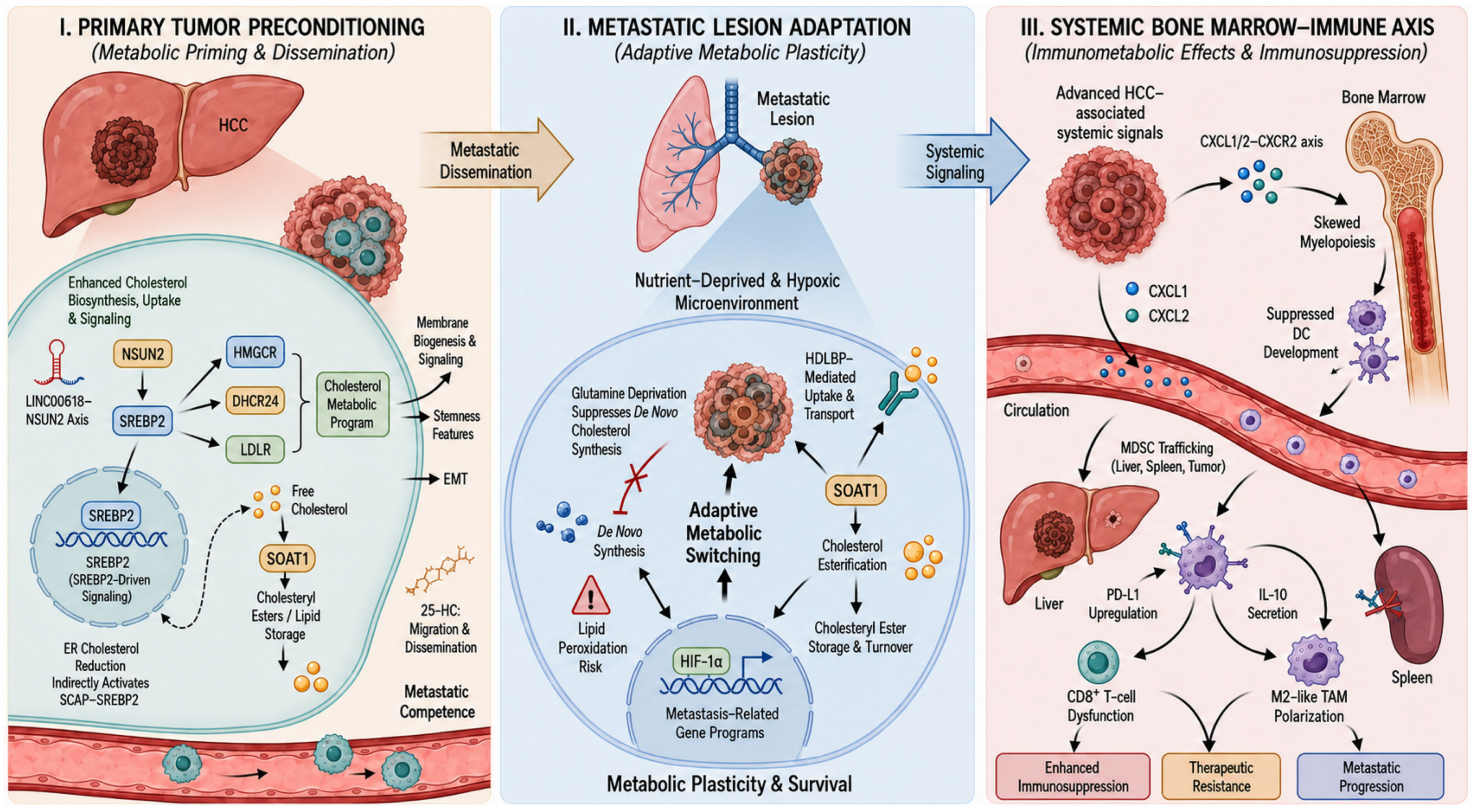
Metastatic HCC couples cholesterol-centered preconditioning with adaptive plasticity and systemic immunometabolic remodeling. This schematic illustrates cholesterol-related metabolic reprogramming and immune remodeling during hepatocellular carcinoma (HCC) metastasis. In the primary tumor, HCC cells with enhanced cholesterol biosynthesis, uptake, or cholesterol-derived signaling may acquire premetastatic advantages through mechanisms such as the LINC00618-NSUN2-SREBP2 axis and cholesterol metabolic genes, including HMGCR, DHCR24, and LDLR. SOAT1 is shown as a cholesterol esterification enzyme that supports cholesteryl ester storage and cholesterol homeostasis, rather than as a direct transcriptional target of SREBP2. Cholesterol-derived metabolites, such as 25-hydroxycholesterol, may further promote EMT, migration, and dissemination. In metastatic lesions, tumor cells may adapt to nutrient deprivation and hypoxia by switching among *de novo* cholesterol synthesis, HDLBP-mediated uptake and transport, cholesterol esterification, and cholesteryl ester storage and turnover. Cholesterol accumulation may stabilize HIF-1 and reinforce metastasis-related gene expression, but the prevalence of this axis in human metastatic HCC remains to be validated. Systemically, advanced HCC can release inflammatory signals that expand and mobilize MDSCs, establishing immunosuppression and contributing to therapeutic resistance. HMGCR, 3-hydroxy-3-methylglutaryl-coenzyme A reductase; DHCR24, 24-dehydrocholesterol reductase; LDLR, low-density lipoprotein receptor; HDLBP, high-density lipoprotein binding protein; HIF-1α, hypoxia-inducible factor 1α; MDSCs, myeloid-derived suppressor cells.

HCC metastasis is not a random event. Within the primary tumor, subpopulations with distinct cholesterol-metabolic features may already be primed for dissemination. Throughout metastatic spread, tumor cells display pronounced metabolic plasticity that enables adaptation to hostile new environments. More importantly, liver cancer can reshape systemic immunity by eliciting systemic inflammation and bone-marrow responses, thereby creating permissive conditions for dissemination and outgrowth (**Figure 3**).

### 6.1 Metabolic preconditioning before metastasis

HCC metastasis is accompanied by profound metabolic reprogramming, but the evidence does not support a single uniform metastatic cholesterol program. Direct and preclinical HCC studies support roles for enhanced cholesterol biosynthesis, lipid transport, esterification, and oxysterol signaling in migration, invasion, EMT, and stress adaptation. However, the relative contribution of synthesis, uptake, efflux, and storage appears stage- and context-dependent. Therefore, metastatic preconditioning should be framed as a flexible cholesterol-metabolic state rather than as a fixed “high synthesis-high efflux” phenotype.

#### 6.1.1 Enhanced cholesterol biosynthesis increases metastatic potential

High-biosynthesis subpopulations within primary lesions may exhibit invasive phenotypes because rapid cholesterol replenishment supports membrane biogenesis, membrane fluidity, lipid-raft signaling, and survival during dissemination. Preclinical HCC evidence shows that the long non-coding RNA LINC00618 stabilizes NSUN2 and enhances NSUN2-mediated m5C modification and stability of SREBP2 mRNA, thereby promoting cholesterol biosynthesis, tumor growth, and metastasis (92). DHCR24 acetylation at K254 sustains cholesterol synthesis and is associated with poor survival, and HMGCR upregulation in cancer stem cell-related studies suggests a potential link between high sterol synthesis, stemness, recurrence, and metastasis (69, 93). Nevertheless, whether these markers define a stable metastatic subpopulation in human HCC remains to be validated.

How a high-synthesis state translates into migratory advantage is often linked to EMT, lipid-raft signaling, and oxysterol-mediated communication. Preclinical HCC evidence indicates that SOAT1 positively regulates EMT and that cholesterol-derived 25-HC can promote HCC cell migration and intrahepatic/extrahepatic metastasis (67, 94). This may suggest a cholesterol synthesis/esterification-oxysterol-EMT trajectory, but it should not be interpreted as a universal mechanism because cholesterol can exert opposite effects on migration depending on membrane organization, receptor localization, and metabolic stress.

#### 6.1.2 Context-dependent cholesterol efflux and lipid transport in metastatic potential

Cholesterol efflux and lipid transport may help maintain intracellular cholesterol balance and stress adaptation during metastasis, but their effects on HCC migration and dissemination are controversial. On one hand, animal models indicate that disrupting cholesterol efflux pathways in epithelial progenitor cells can promote tumor growth, suggesting that efflux pathways materially influence tumor behavior (95). On the other hand, one HCC study reported that higher cholesterol restrained migration and metastasis by promoting CD44 localization in lipid rafts (96). HDLBP is clinically associated with HCC metastasis and may participate in cholesterol-induced metastatic signaling, but it should be described as a lipid transport-related factor rather than direct proof that canonical cholesterol efflux drives HCC metastasis (97).

#### 6.1.3 Synergy between high cholesterol synthesis and high cholesterol efflux

At present, evidence supports roles for enhanced cholesterol biosynthesis and HDLBP-associated cholesterol-induced metastatic signaling, but direct evidence for a defined HCC subpopulation with concurrent “high biosynthesis + high efflux” driving metastasis remains insufficient (69, 92, 97, 98). This model should therefore be retained only as a hypothesis that may explain metabolic flexibility in selected tumor-cell states and remains to be validated in human HCC using single-cell multi-omics, spatial metabolomics, and lineage-tracing approaches.

### 6.2 Metabolic plasticity of metastatic lesions

Metastatic lesions are often nutrient-deprived and hypoxic. HCC cells may toggle among cholesterol synthesis, exogenous uptake, esterification, and mobilization of intracellular stores to maintain sterol balance. Under glutamine deprivation, cholesterol synthesis is markedly suppressed in HCC cells; forcing restoration of synthesis under this condition increases cell death, an effect associated with NADPH depletion and enhanced lipid peroxidation (99). This finding provides an important counterexample to the assumption that high cholesterol synthesis is always advantageous. Instead, metabolic switching itself appears to be a key survival strategy and should be considered when designing cholesterol-targeted therapy.

In hypoxic environments, cholesterol accumulation can influence hypoxia-signaling pathways. Preclinical HCC evidence shows that accumulated cholesterol can inhibit ubiquitin-dependent degradation of hypoxia-inducible factor 1 (HIF-1), thereby stabilizing HIF-1, inducing metastasis-related genes, and promoting lung metastasis (100). Conversely, hypoxia may also reprogram lipid metabolism through HIF-1-dependent transcriptional programs (101). Thus, a bidirectional cholesterol-HIF-1 feedback loop may contribute to metastatic adaptation, but its prevalence and therapeutic relevance in human metastatic HCC remain to be validated.

During dissemination and within the heterogeneous metastatic milieu, tumor cells can also use specific molecules to switch from biosynthesis to uptake. HDLBP is associated with metastasis and may promote cholesterol uptake and transport, thereby reducing reliance on endogenous synthesis when exogenous supply is available (97). SOAT1 contributes to homeostasis through cholesterol esterification and positively regulates EMT, potentially buffering transitions among synthesis, uptake, and storage to support invasion and colonization (67). In addition, oncogenic signals such as growth-factor-related pathways can enhance uptake and use of extracellular cholesterol, enabling tumor cells to switch from synthesis to uptake during nutrient fluctuations (102).

### 6.3 Systemic inflammatory and immunometabolic effects

Advanced HCC can emit systemic signals by releasing inflammation-associated cytokines and chemokines that act remotely on the bone marrow to skew myelopoiesis and drive aberrant expansion and mobilization of MDSCs. This systemic immunometabolic remodeling is supported by HCC-related and broader tumor/stress models, but the specific contribution of cholesterol-derived metabolites to bone-marrow reprogramming remains insufficiently defined. Therefore, systemic inflammation-MDSC expansion should be presented as a validated feature of advanced HCC biology, whereas cholesterol-specific marrow programming remains a hypothesis requiring direct clinical validation.

Under sustained inflammation, pro-inflammatory factors such as CXCL1 and CXCL2 in the HCC tumor microenvironment can become systemically elevated and affect the bone-marrow niche (103, 104). In mouse models, chronic-stress paradigms increase serum CXCL1/CXCL2 and, through CXCR2 signaling, enhance bone-marrow myeloid mobilization and MDSC generation (105). Liver-transplantation-associated acute graft injury can also activate the CXCL10/TLR4/MMP14 axis, mobilizing monocytic MDSCs and increasing recurrence risk after transplantation (106). These data support CXCL/CXCR2- and CXCL10/TLR4/MMP14-linked myeloid mobilization, whereas statements that TGF- or CXCR4 directly skew bone-marrow myelopoiesis through specific noncanonical pathways should remain explicitly cautious unless supported by direct HCC evidence (107–110).

Expanded MDSCs can traffic to the liver, spleen, and tumor sites, thereby establishing a systemic immunosuppressive network. Mechanistically, chronic-stress-induced MDSCs can enter the spleen and tumor microenvironment through chemotactic receptors such as CXCR2, upregulate PD-L1, secrete IL-10, and impair CD8+ T-cell function (105). In intermediate-to-late tumor stages, MDSCs may also inhibit T-cell proliferation through arginase- and ROS-related pathways (108, 111). MDSC-driven M2-like macrophage polarization through the CXCL12 pathway has been described as a reinforcing immunosuppressive loop (112, 113), but the degree to which cholesterol metabolites initiate or amplify this systemic circuit in human HCC remains to be validated.

Clinically, MDSC expansion is associated with tumor aggressiveness, poor prognosis, and reduced response to immune checkpoint inhibitors (ICIs), and it may also contribute to resistance to targeted therapies such as sorafenib (111, 114–117). However, whether cholesterol-related metabolic interventions can reliably reverse systemic MDSC-mediated immunosuppression in patients remains unknown. Interventions targeting CXCR2, CCL15-CCR1, or related myeloid-recruitment axes may represent potential strategies for disrupting this cycle, but these should be considered translational hypotheses rather than established clinical approaches.

## 7 Diagnostic and therapeutic strategies from a dynamic perspective

HCC develops along a continuum from chronic liver disease, with cholesterol-metabolic rewiring operating throughout but varying across stages. Dynamic integration of metabolomic readouts and core gene signatures may support stratification of high-risk individuals, early noninvasive detection, evaluation of tumor aggressiveness and prognosis, and stage-aligned metabolic–immune combination therapies.

### 7.1 Stage-specific biomarkers

During HCC progression, cholesterol-metabolic dysregulation undergoes continuous transitions. A dynamic framework integrating metabolite profiles with key gene signatures may support early risk prediction and, at intermediate-to-late stages, molecular subtyping and prognostic evaluation (**Table 1**).

**Table 1:** Stage-specific biomarker candidates related to cholesterol metabolic reprogramming in HCC.

Disease stage	Representative biomarkers	Potential application	Refs.
High-risk/preneoplastic stage	Taurine-conjugated bile acids; choline-derived metabolites; lipid depletion and polyamine elevation; DHCR24, FDFT1, SOAT1, SCAP, HDLBP, LDLR, CSNK2A1 and AMD1.	Risk warning before radiologic HCC detection; multi-omic stratification of cirrhosis or MASLD/MASH populations; candidate therapeutic guidance.	(67–69, 97, 118–127)
Tumor initiation/early HCC	VLDL-related metabolites; reduced conventional lipid indices; TCA/TCDCA; decreased long-chain acylcarnitines; SQLE, DHCR24/K254ac, ENO1/BACE2-HMGCR/LDLR, SCAP-SREBP, CSNK2A1, LINC00618 and SOAT1.	Noninvasive early diagnosis, recurrence-risk assessment and tissue-based metabolic subtyping.	(67, 69, 92, 119, 121–123, 125, 128–133)
Advanced/metastatic HCC	*27-HC* cholesteryl-ester remodeling; spermine; SOAT1, DHCR24/K254ac, SCAP, HDLBP, FOXK2 and LINC00618.	Assessment of metastatic propensity, therapeutic resistance and prognosis; basis for longitudinal liquid-biopsy monitoring.	(67, 69, 92, 97, 121, 134–137)

HCC, hepatocellular carcinoma; MASLD, metabolic dysfunction-associated steatotic liver disease; MASH, metabolic dysfunction-associated steatohepatitis; TCA, taurocholic acid; TCDCA, taurochenodeoxycholic acid; VLDL, very-low-density lipoprotein; LCACs, long-chain acylcarnitines; DHCR24, 24-dehydrocholesterol reductase; FDFT1, farnesyl-diphosphate farnesyltransferase 1; SOAT1, sterol O-acyltransferase 1; SCAP, SREBP cleavage-activating protein; HDLBP, high-density lipoprotein binding protein; LDLR, low-density lipoprotein receptor; CSNK2A1, casein kinase 2 alpha 1; AMD1, adenosylmethionine decarboxylase 1; SQLE, squalene epoxidase; 27-HC, 27-hydroxycholesterol; MVI, microvascular invasion.

#### 7.1.1 The preneoplastic stage and high-risk background phase

In preneoplastic lesions and high-risk background states, aberrant cholesterol metabolism is associated with malignant transformation. Oxysterols reflect both oxidative stress and cholesterol turnover. In high-risk populations, circulating bile acid profiles and choline-derived metabolites currently have more consistent prospective support as early warning signals for HCC development than a generalized combination of 4-hydroxycholesterol and 25-hydroxycholesterol across HCV and MASLD settings (118). Serum metabolomic profiling suggests that progression from cirrhosis to HCC entails systemic metabolic reprogramming: tauro-conjugated bile acids are increased, whereas acyl-cholines, deoxycholate derivatives, and glycerophosphocholine-related lysophospholipids are decreased; amino-acid/polyamine-related alterations are also observed during progression (119, 120). Combining oxysterols with these features may enable risk warning before radiologic detection.

At the gene level, HCC tissues and some high-risk metabolic background models show directional shifts in cholesterol metabolic pathways. Genes involved in biosynthesis and esterification—such as DHCR24, FDFT1, and SOAT1—are upregulated, supporting cholesterol accumulation and malignant progression (67–69). Cholesterol sensing and transport genes are also perturbed: SCAP overexpression is associated with cholesterol deposition and sorafenib resistance (121); high HDLBP expression correlates with metastasis (97); and LDLR downregulation may further reinforce biosynthetic metabolism (122). There is crosstalk between cholesterol metabolism and immunity: cholesterol can directly activate CSNK2A1 to drive progression of an “oncogenic-cholesterol” subtype (AB-HCC), and elevated AMD1 correlates with immunosuppression and may modulate responsiveness to immunotherapy (123, 124).

Translationally, integrating serum metabolomic signatures such as lipid depletion and polyamine elevation with transcriptomic data may enable the construction of multi-omic risk models to identify high-risk individuals likely to progress from cirrhosis to HCC (125, 126). These markers may further serve as therapeutic guides: CSNK2A1 is a candidate target in AB-HCC; SCAP may help anticipate sorafenib resistance and inform cholesterol-depleting combination strategies; and AMD1 and related polyamine-pathway factors may serve as prognostic markers or therapeutic targets, although their value for predicting immune checkpoint blockade efficacy in HCC still requires further validation (121, 123, 127).

#### 7.1.2 Tumor initiation and early-stage HCC

During tumor initiation and early-stage HCC, cholesterol-metabolic dysregulation contributes more directly to proliferation, survival, and microenvironmental remodeling; accordingly, its metabolite profiles and gene signatures may be leveraged for early diagnosis and risk stratification (128).

At the metabolite level, biofluid-based testing offers a noninvasive option. Lipidomic profiling shows that VLDL-related metabolites are increased in patients with HCC and correlate with progression (129), whereas conventional lipid indices such as total cholesterol and LDL are often decreased (130). Multiple studies have reported increased circulating taurine-conjugated bile acids (e.g., TCA and TCDCA) in HCC, changes that can occur years before diagnosis and are not fully dependent on liver-function indices (119, 131). In addition, baseline plasma long-chain acylcarnitines (LCACs) are decreased in HCC, a change that can be detected before malignant transformation and is linked to impaired mitochondrial fatty-acid oxidation (FAO) (132). Mass-spectrometry-based composite metabolite panels covering lipids, amino acids, choline derivatives, and related metabolites have shown predictive value for early HCC and postoperative recurrence risk (125).

At the gene level, cholesterol biosynthesis and esterification pathways are upregulated early. SQLE is highly expressed in MASLD-HCC transcriptomes, and its protumorigenic effects are associated with cholesteryl-ester accumulation (133). DHCR24 is increased in HCC, and acetylation at K254 is an independent indicator of poor prognosis (69). ENO1 and BACE2 can upregulate cholesterol-biosynthetic genes such as HMGCR and, together with low LDLR expression, promote disease progression (122). The SCAP–SREBP axis is frequently dysregulated; SCAP is overexpressed in sorafenib-resistant HCC and drives cholesterol deposition and resistance (121). Regarding regulatory factors, CSNK2A1 can be directly bound and activated by cholesterol in cholesterol-enriched subtypes, promoting tumorigenesis and associating with an immunosuppressive microenvironment (123); LINC00618 promotes growth and metastasis (92); and SOAT1 maintains cholesterol homeostasis and positively regulates EMT to drive invasion (67). These gene features can be used to subtype tissue specimens and can be jointly evaluated with biofluid metabolomics.

#### 7.1.3 Advanced and metastatic-stage HCC

In advanced and metastatic stages, cholesterol-metabolic reprogramming is more prominently manifested as increased invasiveness, drug resistance, and poor prognosis. At the metabolite level, elevated 27-HC is associated with metastasis and may indicate the establishment of a prometastatic microenvironment (134). Cholesteryl-ester remodeling is evident in advanced HCC, but the direction of change is context-dependent rather than uniformly decreased across serum and tumor tissue (135). Increased arginine-metabolism-related products such as spermine are associated with invasive phenotypes and heightened microenvironmental heterogeneity and may therefore help assess metastatic propensity (136).

At the gene level, SOAT1 is associated with EMT and metastatic risk, whereas DHCR24, including K254 acetylation, is elevated in HCC and independently predicts shortened survival (67). SCAP overexpression is linked to sorafenib resistance; it is overexpressed in sorafenib-resistant HCC cells and tissues and drives cholesterol deposition, making it a potential marker for therapeutic-resistance assessment (121). HDLBP, FOXK2, and LINC00618 are also associated with metastasis and invasive potential (92, 97, 137). Clinically, these markers can be integrated with microvascular invasion (MVI), histological grade, and other features to build multidimensional risk frameworks, and liquid-biopsy approaches may be explored for longitudinal monitoring.

### 7.2 Stage-specific therapeutic interventions

Across the continuum from chronic liver disease to metastatic dissemination, the metabolic features of liver cancer continually evolve. Accordingly, intervention strategies should be designed in a stage-specific manner within an “early blockade-targeted eradication-late-stage breakthrough” framework. Importantly, the evidence level differs across interventions: statins are supported mainly by observational associations with HCC risk reduction; FXR agonists have clinical-trial evidence for MASH/NASH fibrosis but not definitive HCC-prevention proof; SQLE-targeted strategies are supported largely by preclinical MASH-HCC models; and advanced-stage metabolic combinations remain biomarker-guided hypotheses requiring prospective testing (**Table 2**).

**Table 2:** Stage-specific therapeutic opportunities targeting cholesterol-related vulnerabilities in HCC.

Disease stage	Therapeutic focus	Representative strategies and evidence	Refs.
Chronic liver disease	Prevention and interception	FXR agonists for MASH fibrosis; statins as cholesterol-synthesis inhibitors with observational links to lower HCC risk; weight control, glycemic/lipid management and antifibrotic care remain foundational.	(33–38, 138–141)
Early HCC	Precision eradication of cholesterol-high lesions	Target SQLE-high, cholesterol-biosynthetic tumor states. Terbinafine/SQLE inhibition may relieve cholesterol-driven immunosuppression, restore CD8+ T-cell activity and synergize with anti-PD-1 in preclinical MASH-HCC models.	(56, 133)
Advanced/metastatic HCC	Overcoming metabolic adaptation	Use biomarker-guided combinations to block compensatory metabolism, including glucose uptake plus mitochondrial function, lipogenesis plus MAPK signaling, metabolic-epigenetic targeting and metabolic-immunotherapy combinations.	(142–146)

HCC, hepatocellular carcinoma; FXR, farnesoid X receptor; MASH, metabolic dysfunction-associated steatohepatitis; SQLE, squalene epoxidase; PD-1, programmed cell death protein 1; MDSCs, myeloid-derived suppressor cells; MAPK, mitogen-activated protein kinase; ICI, immune checkpoint inhibitor.

#### 7.2.1 Prevention and interception (chronic liver disease stage)

The goal of intervention during chronic liver disease is to reduce HCC risk and halt fibrotic progression. The bile acid–FXR axis links cholesterol metabolism to the regulation of inflammation and fibrosis. FXR agonists have shown histological signals of benefit in clinical trials for MASH fibrosis, but adverse effects such as pruritus and lipid alterations, as well as long-term outcomes, require further validation (138–140).

Statins reduce cholesterol biosynthesis by inhibiting HMG-CoA reductase and also exert pleiotropic effects, including anti-inflammatory actions and improved endothelial function. Multiple cohort studies and systematic reviews suggest that statin use is associated with reduced HCC risk, with particularly consistent findings in populations with MASLD and viral hepatitis (141). Because this evidence is derived predominantly from observational studies, confounding and indication bias should be carefully considered; clinically, statins should still be prescribed primarily for cardiovascular benefit, with monitoring guided by hepatic function and potential drug–drug interactions.

Beyond pharmacotherapy, weight management, glycemic and lipid control, and integrated anti-inflammatory/antifibrotic interventions constitute foundational strategies during the chronic liver disease stage. Quantifying their effects on HCC risk will still require long-term follow-up and prospective real-world studies.

#### 7.2.2 Precision eradication (early HCC)

A central objective in early HCC is to delineate metabolic heterogeneity and target the subpopulation with high cholesterol-biosynthetic activity. High SQLE expression can drive aberrant cholesterol biosynthesis and contribute to an immunosuppressive microenvironment. SQLE inhibitors such as terbinafine suppress the activity of this key enzyme and, in preclinical MASH-HCC models, alleviate cholesterol-driven immunosuppression, restore CD8+ T-cell activity, and reduce suppressive myeloid cells such as Arg-1+ MDSCs. Combination with anti-PD-1 therapy yields synergistic effects, resulting in stronger tumor-growth suppression *in vivo* (56).

#### 7.2.3 Overcoming adaptation (advanced HCC)

Drug resistance in advanced HCC is closely linked to metabolic plasticity. Tumor cells can switch to alternative pathways when a given route is blocked and can reprogram metabolism under drug pressure; metabolites may also participate in epigenetic regulation, thereby altering the expression of resistance-related genes (142–144). To address this feature, multi-node combination blockade can be considered: inhibiting glucose uptake while simultaneously suppressing mitochondrial function to disrupt ATP supply (142); pairing enhanced lipogenesis with MAPK-pathway inhibition to induce synthetic lethality (145); combining metabolic and epigenetic interventions to increase drug sensitivity (146); or integrating metabolic targeting with immunomodulation to improve the efficacy of immune checkpoint inhibitors through microenvironmental remodeling (142). Future directions include stratified combination therapies guided by metabolic subtypes, simultaneous targeting of metabolic dependencies in both tumor cells and myeloid cells, and the use of delivery technologies and biomarker monitoring to improve safety and controllability.

## 8 Discussion

### 8.1 Summary of core conclusions

This review highlights cholesterol-metabolic rewiring as a dynamic and context-dependent process during hepatocarcinogenesis. Rather than representing a uniform increase in cholesterol metabolism, HCC progression involves stage- and cell-type-specific changes in cholesterol synthesis, uptake, esterification, efflux, storage, and conversion. In chronic metabolic liver disease, free-cholesterol loading, impaired bile acid-FXR feedback, oxysterol accumulation, and SREBP2-SCAP dysregulation may connect hepatocyte stress with HSC activation, macrophage remodeling, inflammation, and fibrosis. In preneoplastic lesions and early HCC, cholesterol biosynthetic and esterification programs may support membrane remodeling, lipid-raft-associated oncogenic signaling, metabolic autonomy, and impaired immune surveillance. In advanced and metastatic HCC, metabolic plasticity and spatial heterogeneity appear to link cholesterol-active tumor regions with exhausted T cells, TAMs, MDSCs, extracellular vesicle signaling, and oxysterol-mediated intercellular communication.

### 8.2 Limitations of existing evidence

Several limitations should be emphasized. First, many proposed mechanisms are supported by HCC cell lines, mouse models, diet-induced MASH-HCC models, or non-HCC tumor systems, whereas direct validation in human HCC tissues remains incomplete. Second, the biological effects of cholesterol are highly context-dependent. For example, cholesterol biosynthesis may promote tumor growth and immune escape in some settings, whereas under nutrient stress or high-fatty-acid conditions, suppression or restoration of cholesterol synthesis may produce divergent outcomes. Third, immune effects should be interpreted as cholesterol dyshomeostasis rather than simple cholesterol excess, because both cholesterol accumulation and oxysterol-driven cholesterol deficiency in immune cells can impair antitumor immunity. Fourth, HCC is etiologically heterogeneous; mechanisms identified in MASLD/MASH-HCC may not be directly transferable to HBV-, HCV-, alcohol-, or toxin-related HCC without comparative validation.

### 8.3 Technical bottlenecks

Current technologies still limit causal interpretation of cholesterol-immune crosstalk. Single-cell RNA sequencing provides cellular resolution but incompletely captures lipid abundance, cholesterol flux, and oxysterol diversity. Spatial transcriptomics can identify metabolic-immune neighborhoods but cannot by itself prove metabolite exchange or pathway activity. Spatial metabolomics and imaging mass spectrometry offer direct lipid readouts, yet their resolution, sensitivity, metabolite annotation, and compatibility with clinical specimens remain technically challenging. In addition, cholesterol and oxysterols are sensitive to sample handling, oxidation, storage conditions, and extraction methods, making cross-cohort comparison difficult. Therefore, future studies should integrate spatial transcriptomics, spatial proteomics, lipidomics, stable-isotope tracing, and functional perturbation assays.

### 8.4 Challenges in clinical translation

The translation of cholesterol-targeted strategies into HCC management faces several challenges. Statins are associated with reduced HCC risk in observational studies, but they should currently be regarded primarily as cardiovascular-risk therapies with potential hepatic benefit rather than established antitumor agents. FXR agonists have shown antifibrotic signals in MASH/NASH trials, but adverse effects, lipid alterations, and long-term cancer-prevention outcomes require further evaluation. Direct targeting of SQLE, SOAT1, SCAP, DHCR24, or related nodes may offer therapeutic opportunities, but patient selection, toxicity, compensatory metabolic rewiring, and combination timing remain unresolved. For advanced HCC, metabolic targeting is unlikely to be effective as a uniform strategy; biomarker-guided combinations with immune checkpoint blockade, anti-angiogenic therapy, epigenetic therapy, or myeloid-cell modulation will require prospective validation.

### 8.5 Future research directions

Future research should move in four directions. First, spatial multi-omics studies should map cholesterol-related metabolites, immune checkpoints, macrophage states, and stromal programs within the same tissue sections to define clinically relevant metabolism-immune niches. Second, patient-derived organoids, tumor spheroids, organoid-immune co-culture systems, and liver-on-chip models should be used to test whether cholesterol-related pathways directly reshape immune-cell recruitment and function. Third, longitudinal clinical cohorts should compare MASLD/MASH-, HBV-, HCV-, alcohol-, and mixed-etiology HCC, integrating serum lipidomics, bile acid profiles, oxysterols, tumor transcriptomics, spatial pathology, recurrence, metastasis, and immunotherapy response. Fourth, drug-translational studies should evaluate cholesterol-targeting agents in biomarker-defined populations and determine rational combinations, optimal treatment windows, safety profiles, and resistance mechanisms. These approaches will help determine whether cholesterol-metabolic rewiring is merely a disease-associated phenotype or a clinically actionable driver of HCC progression.
